# Distinct Skeletal Muscle Gene Regulation from Active Contraction, Passive Vibration, and Whole Body Heat Stress in Humans

**DOI:** 10.1371/journal.pone.0160594

**Published:** 2016-08-03

**Authors:** Michael A. Petrie, Amy L. Kimball, Colleen L. McHenry, Manish Suneja, Chu-Ling Yen, Arpit Sharma, Richard K. Shields

**Affiliations:** 1 Department of Physical Therapy and Rehabilitation Science, Carver College of Medicine, The University of Iowa, Iowa City, Iowa, United States of America; 2 Department of Internal Medicine, Carver College of Medicine, The University of Iowa, Iowa City, Iowa, United States of America; 3 Department of Biochemistry, Carver College of Medicine, The University of Iowa, Iowa City, Iowa, United States of America; 4 Department of Veterans Affairs, VA Medical Center, Iowa City, Iowa, United States of America; Cinvestav-IPN, MEXICO

## Abstract

Skeletal muscle exercise regulates several important metabolic genes in humans. We know little about the effects of environmental stress (heat) and mechanical stress (vibration) on skeletal muscle. Passive mechanical stress or systemic heat stress are often used in combination with many active exercise programs. We designed a method to deliver a vibration stress and systemic heat stress to compare the effects with active skeletal muscle contraction. ***Purpose*:** The purpose of this study is to examine whether active mechanical stress (muscle contraction), passive mechanical stress (vibration), or systemic whole body heat stress regulates key gene signatures associated with muscle metabolism, hypertrophy/atrophy, and inflammation/repair. ***Methods*:** Eleven subjects, six able-bodied and five with chronic spinal cord injury (SCI) participated in the study. The six able-bodied subjects sat in a heat stress chamber for 30 minutes. Five subjects with SCI received a single dose of limb-segment vibration or a dose of repetitive electrically induced muscle contractions. Three hours after the completion of each stress, we performed a muscle biopsy (vastus lateralis or soleus) to analyze mRNA gene expression. ***Results*:** We discovered repetitive active muscle contractions up regulated metabolic transcription factors NR4A3 (12.45 fold), PGC-1α (5.46 fold), and ABRA (5.98 fold); and repressed MSTN (0.56 fold). Heat stress repressed PGC-1α (0.74 fold change; p < 0.05); while vibration induced FOXK2 (2.36 fold change; p < 0.05). Vibration similarly caused a down regulation of MSTN (0.74 fold change; p < 0.05), but to a lesser extent than active muscle contraction. Vibration induced FOXK2 (p < 0.05) while heat stress repressed PGC-1α (0.74 fold) and ANKRD1 genes (0.51 fold; p < 0.05). ***Conclusion*:** These findings support a distinct gene regulation in response to heat stress, vibration, and muscle contractions. Understanding these responses may assist in developing regenerative rehabilitation interventions to improve muscle cell development, growth, and repair.

## Introduction

Skeletal muscle is an important regulator of overall systemic health and well-being. Genes for physical activity have been highly conserved, as mobility, function, and human performance have been key components of survival. With the emergence of automated transportation methods (car, plane, train), and lifestyle altering technologies (television, computers, and cell phones), the primary regulator of human skeletal muscle, physical activity, has declined. The impact of this decline on healthy people, with less than optimal lifestyle choices, is profound and contributes to an obesity epidemic[1].

Often overlooked is the impact of reduced physical stress on people who are disabled [2]. People with a central nervous system (CNS) injury, who are unable to activate their muscles completely, suffer from a host of systemic co-morbidities with a known link to reduced skeletal muscle activity, including diabetes and osteoporosis [3–6]. In this report, we explore viable rehabilitative interventions to understand the external factors that influence skeletal muscle. Specifically, we compare three forms of stress including actively induced muscle contraction, passive mechanical vibration, and whole body heat stress on skeletal muscle gene regulation.

Active mechanical stress, through muscle contraction, is a powerful stimulus to skeletal muscle, however, this form of stress also has important influences on systemic metabolic flexibility. Skeletal muscle is capable of regulating up to 75% of the body’s metabolism of glucose [7]. Regular muscle activation triggers cellular mitochondrial biogenesis and regulates key molecular pathways associated with muscle cell metabolism, muscle cell hypertrophy, and muscle cell regeneration [8, 9]. The high incidence of diabetes in people with paralysis underscores that skeletal muscle activity is important to the systemic health of people with a spinal cord injury [10–12]. Inducing “active” mechanical stress through neuromuscular electrical stimulation may offer an alternative for people with paralysis to improve their systemic metabolic health through regular muscle activity [9, 13–18].

Another form of mechanical stress is vibration. Applying specific frequencies of vibration (20–50 Hz) at a given gravitational force (g force; 0.3 to 0.6) regulates musculoskeletal plasticity in animal models [19, 20] and spinal cord excitability in humans [21, 22]. Recent reports suggest that “cross-talk” between skeletal muscle and the underlying bone raises the possibility that skeletal muscle and bone communicate through common pathways [23]. If mechanical inputs (vibration) also regulate muscle metabolic/hypertrophic signaling pathways, then vibration may be complementary to active contractions; notably a potential influences on people who have limited capacity to activate their own muscles because of a compromised central nervous system (CNS).

A third form of stress is whole body heat stress [24]. Increasing core body temperature is a natural consequence of whole body exercise and likely coordinates signaling among all tissues (brain, heart, muscle, liver, skin). The ability to lose body heat through sweating is a unique and well-conserved function that is essential in sustaining repetitive skeletal muscle activity[25]. The close association between increased core body temperature and skeletal muscle activity suggests that these systems share common signaling pathways[26]. From previous studies, we know that passive whole-body heat stress does not increase skeletal muscle temperature, but does increase systemic sympathetic drive as well as several blood biomarkers; catecholamines, heat shock proteins, and serotonergic-dopaminergic precursors [27]. The increased extracellular biomarkers from passive heat stress may regulate several systemic responses, including glucose tolerance [28–31]. The impact of whole body heat stress on skeletal muscle signaling, therefore, has important translational implications for people who lose the ability to exercise and sweat through CNS injury (i.e. people with quadriplegia).

The comparative role of three distinct forms of stress (active contraction, passive vibration, and systemic heat stress) on skeletal muscle signaling remains unknown. The methodologies developed for this study test two novel stresses: vibration and whole body heat stress on skeletal muscle as compared to a dose of electrically induced active muscle contraction.

Our purpose is to examine whether active mechanical stress (muscle contraction), passive mechanical stress (vibration), or systemic whole body environmental stress (heat) modulates key genetic signatures associated with skeletal muscle metabolism, oxidative pathways, mitochondrial biogenesis, and hypertrophy in human skeletal muscle. Secondarily, we examine the most regulated genes to determine the extent of shared signaling. We expect that active muscle stress will be most effective at regulating skeletal muscle metabolic gene signatures, but that other forms of stress will play key regulatory roles. In addition, we expect to discover overlap among genes that are most regulated in response to each distinct stress condition.

## Methods

### Subjects

Eleven subjects, six able-bodied and five with chronic spinal cord injuries (SCI), participated in the study (Table 1). Five subjects with a SCI received a single dose of limb-segment vibration 3 hours before a percutaneous soleus muscle biopsy (SCI 1–5). Three SCI subjects also received a single session of muscle activity using neuromuscular electrical stimulation of the soleus 3 hours before a percutaneous soleus muscle biopsy (SCI 2, 4, and 5). There was at least a three-month gap between the vibration and neuromuscular electrical stimulation interventions. Six able-bodied subjects sat in a whole-body heat stress chamber for 30 minutes prior to a vastus lateralis muscle biopsy. We opted to use able-bodied subjects for the whole-body heat stress because people with SCI cannot sweat. We focused on the VL muscle, because the VL shows a phenotype that is similar to the chronically paralyzed soleus muscle [32, 33]. Pilot studies in our lab support that gene regulation of the healthy VL was highly correlated (0.91) to that of the paralyzed soleus muscle during an identical electrical stimulation protocol. Importantly, the contractile speeds between the VL and paralyzed soleus muscle are similar (~70–80 ms time to peak) as compared to the healthy soleus (~150 ms time to peak).

**Table 1:** Subject Demographics.

Subject	Sex	Age (Yrs.)	Injury Level	Time Post Injury (Yrs.)	Height (in)	Weight (lbs)	Body Mass Index	Body Fat (%)
AB1	Male	38	-	-	68.5	161.2	24.2	14.9
AB2	Male	38	-	-	76	201	24.5	19.5
AB3	Male	40	-	-	69	202.4	29.9	28.8
AB4	Male	27	-	-	70.5	167.8	23.7	17.2
AB5	Male	27	-	-	73	234.8	31	30.8
AB6	Male	26	-	-	73	133.4	17.6	6.5
SCI1	Male	32	T9	10	72	130	17.6	-
SCI2	Male	39	T10	2	72	230	31.2	-
SCI3	Male	24	T8	4	76	155	17.9	-
SCI4	Male	30	T10	4	73	170	22.4	-
SCI5	Male	28	T10	2	69	251	37.1	-

SCI and AB indicate those subjects with or without a spinal cord injury, respectively.

Our sample size of five for vibration, three for electrical stimulation, and six for heat stress provided us with over 80% power to detect a 50% change in metabolic gene regulation. We normalized all gene regulation values to the opposite limb, which reduced variation from different diets, age, weight, and socioeconomic differences. Because of our within subject design we had excellent power with few subjects. As such, we normalized all genes to the identical genetics of a limb that did not see the muscle contraction, vibration, or whole-body heat stress condition. Prior to enrollment, all subjects provided written consent approved by the University of Iowa Institutional Review Board in accordance with the Helsinki Declaration.

### Active Mechanical Stress: Muscle contraction

**Fig 1:**
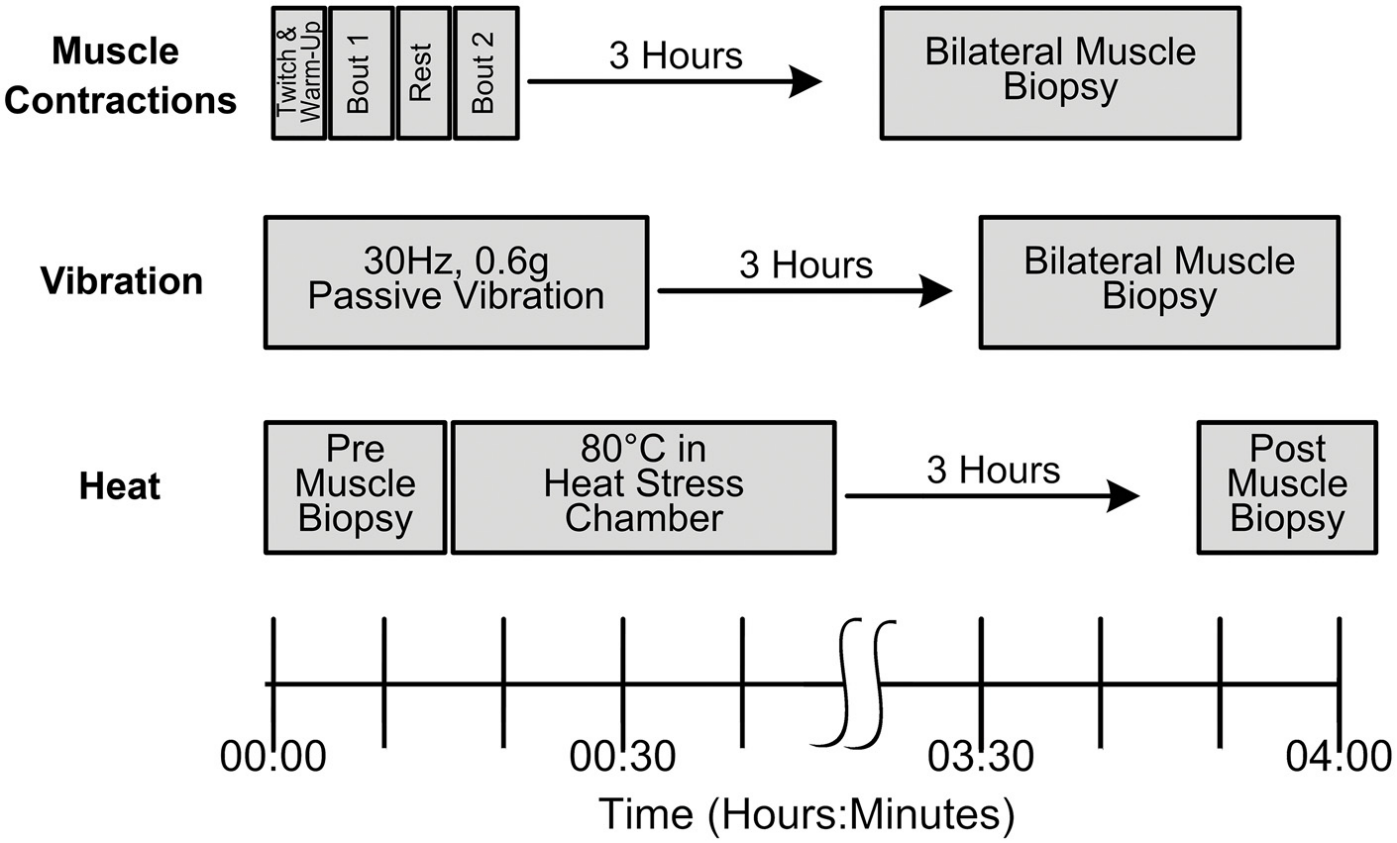
Study Timeline. The muscle contraction stressor was unilaterally delivered in two ~4-minute bouts with a 5-minute rest period between bout 1 and bout 2. The vibration stressor was unilaterally delivered in a single 30-minute bout at a oscillation frequency of 30Hz and an amplitude of 0.6g. The whole-body heat stressor was delivered in a single 30-minute bout. Three hours after the completion of each stressor a percuatneous muscle biopsy was performed. Muscle biopsies were performed bilaterally on the experimental and control limb for the muscle contractions and vibration stressors. A unilateral muscle biopsy was performed before the heat stressor on the control limb and after the heat stressor on the opposite limb due to the systemic effects of whole-body heat stress.

We delivered a dose of active mechanical stress using electrical muscle stimulation to elicit unilateral soleus muscle contractions. Subjects sat in a wheelchair with one leg placed in a testing apparatus to elicit isometric contractions of the plantar flexors using a previously reported protocol [9, 34]. The ankle and knee were flexed to 90°and secured to the apparatus with soft straps above the knee. Self-adhesive carbon electrodes were placed over the plantar flexors and stimulation was provided by a computer-controlled constant current electrical stimulator with a 0 to 400—milliamp range at 400 volts (Digitimer Model DS7A, Digitimer Ltd., Welwyn Garden City, and Hertfordshire, England). Single pulses were given at increasing intensity until maximal twitch torque was observed via an oscilloscope. Stimulation intensity was increased an additional 50% and remained at this level for the remainder of the experiment to obtain supra maximal activation. Subjects received five warm-up contractions (10 Hz, 7 pulses per contraction) to potentiate the plantar flexor muscles and minimize the risk of muscle strain. After a warm-up bout, subjects received seven stimulus pulses using a 10 Hz stimulation train. We delivered 120 isometric contractions using a one on two off work-rest ratio. After a 5-minute recovery period, subjects received a second bout of 120 contractions at the same intensity and frequency. Three hours after the completion of the bout of muscle activity, the experimental and control limbs underwent a percutaneous muscle biopsy (Fig 1).

### Passive Mechanical Stress: Limb-segment Vibration

We delivered a dose of passive mechanical stress unilaterally using a custom vibration apparatus applied to the distal leg [21, 22, 35]. The custom apparatus secured the distal leg to a servo-controlled vibration generator (Ling Dynamic Systems, Royston, Herts, and England). Subjects were positioned so the distal leg was secured to the vibration platform with the heel centered on the vibration platform and the hip, knee, and ankle flexed to 90°. Waist and chest straps were secured to the mounted seat for support. In this position, the vibratory stress was delivered to the lower limb with limited transmissibility to the opposite limb, trunk, and head[35]. A computer controlled vibration platform delivered an oscillation frequency of 30Hz at an acceleration of 0.6g for 30 minutes. Three hours after the completion of the bout of vibration, the experimental and control limbs underwent a percutaneous muscle biopsy (Fig 1).

### Systemic Environmental Stress: Whole-Body Heat Stress

We delivered a dose of systemic environmental stress using a specially instrumented, custom designed low humidity (20%) heat stress chamber set at a temperature of ~73°C [27]. Subjects sat upright in the heat stress chamber for 30 minutes. Subjects sat passively, with minimal movement of the lower extremities (Fig 1). We monitored heart rate, temperature, and thermal comfort for each subject. After 30-minutes of passive sitting, we re-weighed, rehydrated with water, and kept the subject supine at room temperature. Three hours after exiting the heat stress chamber, we performed a unilateral muscle percutaneous muscle biopsy. We performed a control muscle biopsy on the opposite limb prior to the heat stress intervention.

### Muscle Biopsy Protocol

Subjects underwent percutaneous muscle biopsies 3 hours after the interventions. The active and passive mechanical stress subjects underwent bilateral soleus muscle biopsies because only one limb received the intervention. The systemic environmental stress subjects underwent unilateral muscle biopsies from one leg 3 prior to the heat stress, and then 3 hours after the whole-body heat stress intervention. Our muscle biopsy procedure has been previously described [8, 9, 17]. Briefly, we took percutaneous muscle biopsies from both the intervention and control limb of each subject using a 14-gauge Temno biopsy needle (T1420, Cardinal Health) under ultrasound guidance within a sterile field. This smaller needle improved our capacity to sample the atrophied soleus muscle. Four passes of the needle were made to assure a wide sampling range within the muscle. Each pass of the needle was through the same incision site, but we altered the needle angle to sample a different part of the muscle. Each pass obtained approximately 20 mg of muscle tissue. Following harvest, muscle samples were placed in RNA Later (Ambion) and stored at -80°C until further use.

### RNA Extraction and Analysis

Our RNA extraction and analysis procedure has been previously described [18]. Briefly, RNA was extracted using the RNAeasy Fibrous Tissue Kit (Qiagen) with DNAse to remove genomic DNA from final samples. Microarray hybridizations were performed at the University of Iowa DNA Facility as previously reported[8, 18]. Briefly, 50 ng total RNA was converted to SPIA amplified cDNA using the WT-Ovation Pico RNA Amplification System, v1 (NuGEN Technologies, San Carlos, CA, Cat.#3300) according to the manufacturer’s recommended protocol. The amplified SPIA cDNA product was purified through a QIAGEN MinElute Reaction Cleanup column (QIAGEN Cat #28204) according to modifications from NuGEN. Four μg of SPIA amplified DNA were used to generate ST-cDNA using the WT-Ovation Exon Module v1 (NuGEN Technologies, Cat #2000) and again cleaned up with the Qiagen column as above. 5μg of this product were fragmented (average fragment size = 85 bases) and biotin labeled using the NuGEN FL-Ovation cDNA Biotin Module, v2 (NuGEN Technologies, Cat. #4200) as per the manufacturer’s recommended protocol. The resulting biotin-labeled cDNA was mixed with Affymetrix eukaryotic hybridization buffer (Affymetrix, Inc., Santa Clara, CA), placed onto Human Exon 1.0 ST arrays (Part No. 900650), and incubated at 45°C for 18 h with 60 RPM rotation in an Affymetrix Model 640 Genechip Hybridization Oven. Following hybridization, the arrays were washed, stained with streptavidin-phycoerythrin (Molecular Probes, Inc., Eugene, OR), signal amplified with anti-streptavidin antibody (Vector Laboratories, Inc., Burlingame, CA) using the Affymetrix Model 450 Fluidics Station. We scanned arrays with the Affymetrix Model 3000 scanner with 7G upgrade and we collected the data using the GeneChip operating software (GCOS) v1.4. We submitted all to the Gene Expression Omnibus (GSE82323) and are MIAME compliant.

The Affymetrix Human Exon 1.0 ST hybridized arrays were normalized using a Robust Multi-array Average (RMA) and transformed into a log_2_ hybridization signal using Partek Genomic Suites (v6.6 2013 Partek Inc., St. Louis, MO, USA). All mRNA transcripts with log_2_ hybridization signals less than 2 standard deviations below the mean signal intensity for all subjects were discarded from the analysis, restricting the analysis to only those mRNA transcripts with high signal relative to background.

We calculated the fold change by taking the ratio of the hybridization signal of the experimental limb relative to the control, non-stressed limb. A within group paired sample t-test was used to determine the expression change between the experimental and control limbs. We only accepted transcripts that had a significant p-value less than 0.05 and a fold change greater than 1.5 or less than 0.667. We report the group (muscle contraction, vibration, and whole-body heat stress) mean ±standard error for a subset of genes previously identified to be associated with oxidative metabolism, muscle hypertrophy, and mitochondrial biogenesis.

## Results

### Physiological Effects of Muscle Contraction, Vibration, and Heat Stress

**Fig 2:**
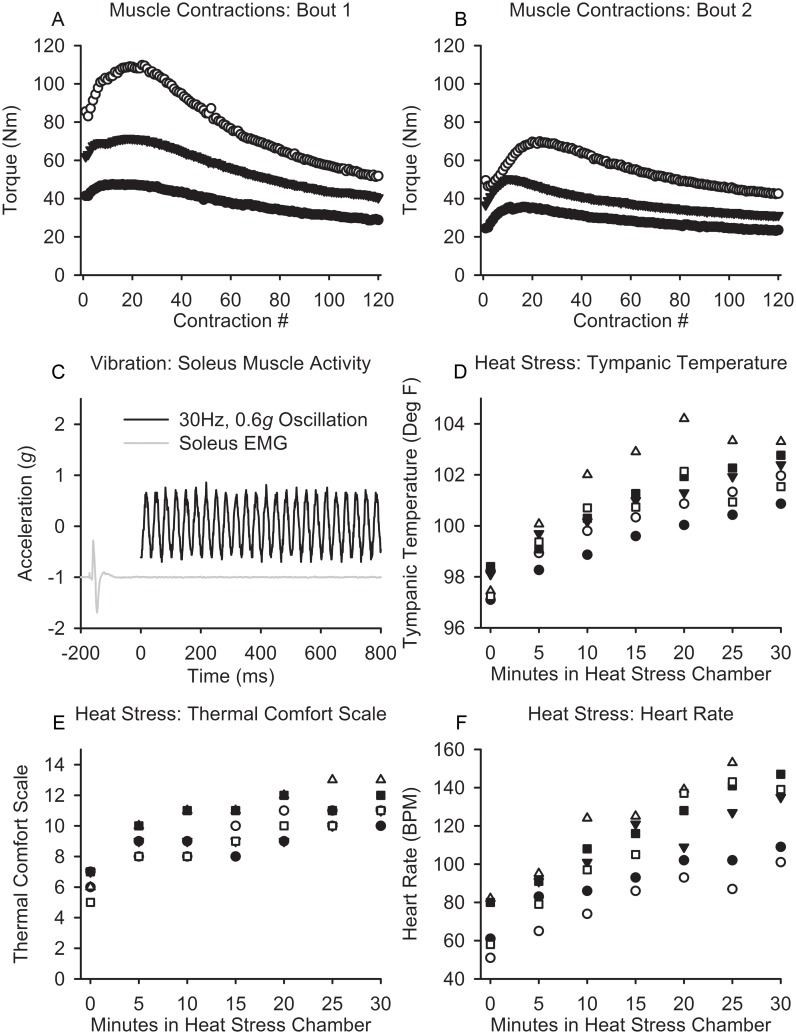
Physiological Effects after Muscle Contraction, Vibration, and Heat. (A and B) The torque curve from the soleus muscle during bout 1 and bout 2 during the muscle contraction stressor with decreased torque production by the end and between the bout 1 and bout 2. Representative example of the level of EMG muscle activity compared to the maximum M-wave during the bout of unilateral muscle vibration. (D, E, F) The temperature, self-reported comfort, and heart rate during the passive, whole-body heat stressor.

The mean peak toque during bout 1 was 75.9±31.0 Nm while the mean peak torque for bout 2 was 50.8±17.5 Nm (p = 0.013). The mean final torque during bout 1 decreased to 40.4±11.5 Nm while the mean final torque in bout 2 decreased to 32.5±9.6 Nm. The magnitude of fatigue supported that we induced a significant physiological challenge to the soleus muscle tissue during active muscle contraction. We illustrate the torque-time curves for two bouts of exercise for three subjects (Fig 2A and 2B).

The vibration stress was purely mechanical as there was no evidence of concurrent soleus muscle activity (EMG activity) during the protocol. Previous investigations in our laboratory support that this vibration intervention inhibits motor neuron pool excitability [22] confirming that the intervention is purely a passive mechanical stress. We present a single subject’s recording showing minimal EMG activity during the vibration protocol (Fig 2C).

The physiological responses to thirty minutes of whole body heat stress at 82 degrees centigrade were robust. The mean heart rate increased from 65.3±12.6 to 132.3±23 (p<0.001), the mean tympanic temperature increased from 97.8±0.7 to 102.1±0.9 (p<0.001), and the mean self-reported thermal scale rating increased from 6.3±0.8 to 11.3±1.0 (p<0.001) (Fig 2D–2F).

Taken together, we were successful in: 1) challenging skeletal muscle through the active contraction protocol as evident from the change in muscle force; 2) delivering a primarily mechanical vibration to the muscle at the prescribed frequency and gravitational force [35] without contamination via active muscle contractions (EMG); and 3) inducing a systemic stress on the cardiovascular system from passive heat stress.

### Skeletal Muscle Signaling Analysis

Of the >15,000 genes analyzed, 1,928, 560, and 2,266 genes had a p-value less than 0.05 for the muscle contraction, vibration, and heat stress groups, respectively, supporting that the intervention changed the regulation of these genes. When we restricted the analysis to only those genes that also had at least a 1.5 fold change, the muscle contraction protocol caused an increase in 240 genes (>1.5 fold change) and a decrease of 17 genes (<0.667 fold change is equivalent to a <-1.5 fold change). Vibration triggered an increase of 13 genes (>1.5 fold change) and a decrease of eight genes (<0.667). Heat stress triggered an increase of 64 genes (>1.5 fold change) and a decrease of 30 genes (<0.667).

**Fig 3:**
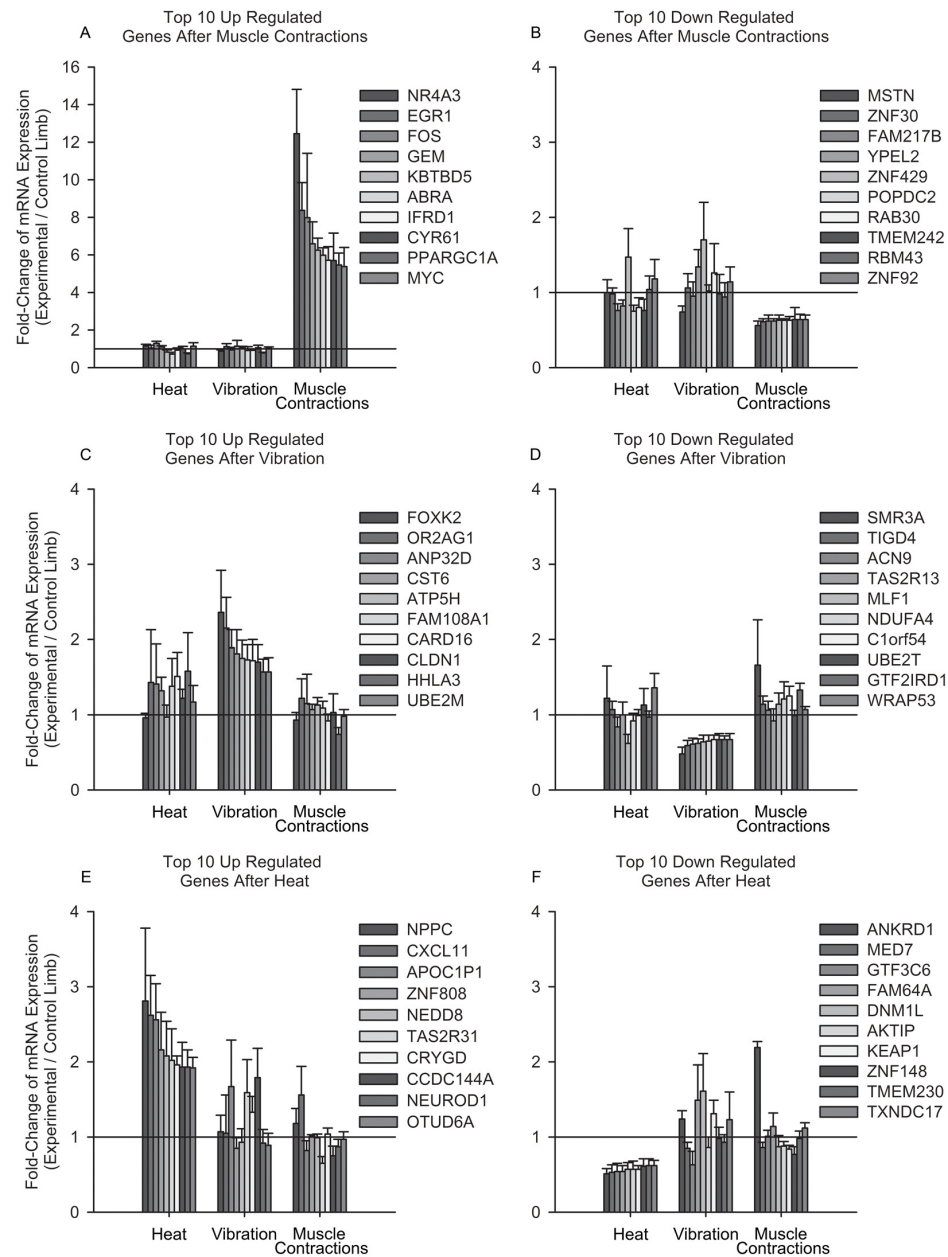
Comparative Gene Stress Responses following Muscle Contractions, Vibration, and Heat. We compared the expression levels of the top 10 up and down regulated genes after each stressor to the other stressors. Of particular note is the order of magnitude change of the top 10 genes increased after muscle contractions compared to the other stressors. Additionally, the increased expression of FOXK2 (2.36±0.56) only after vibration, and the difference in ANKRD1 expression after heat (downregulated, 0.51±0.07) and muscle contractions (upregulated, 2.19±0.08).

We selected the 10 genes with the highest and lowest fold-changes for each group, to compare the expression signature of those genes across all groups (Fig 3). Muscle contractions consistently increased the expression of transcription factors: peroxisome proliferator-activated receptor gamma, coactivator 1 alpha(PGC-1α), nuclear receptor subfamily 4 group A member 3(NR4A3), interferon-related developmental regulator 1(IFRD1), actin binding Rho activating protein(ABRA), early growth response 1(EGR1), and myostatin (MSTN). These genes are linked to regulating oxidative metabolism (PGC-1α and NR4A3), mitochondrial dynamics (IFRD1), and muscle hypertrophy (ABRA and EGR1), while suppressing a potent regulator of muscle atrophy (MSTN).

Passive vibration and whole-body heat stress resulted in smaller fold-changes compared to the gene expression changes observed after muscle contractions. Forkhead box K2 (FOXK2) was increased after vibration. FOXK2 is a potent regulator for Wnt signaling proteins. Three hours after whole-body heat stress ankyrin repeat domain 1(ANKRD1) expression was decreased compared to an increased expression after muscle contractions. Most of the other genes with altered expression after whole-body heat stress have poorly defined roles in human skeletal muscle.

### Metabolic Gene Profile

**Fig 4:**
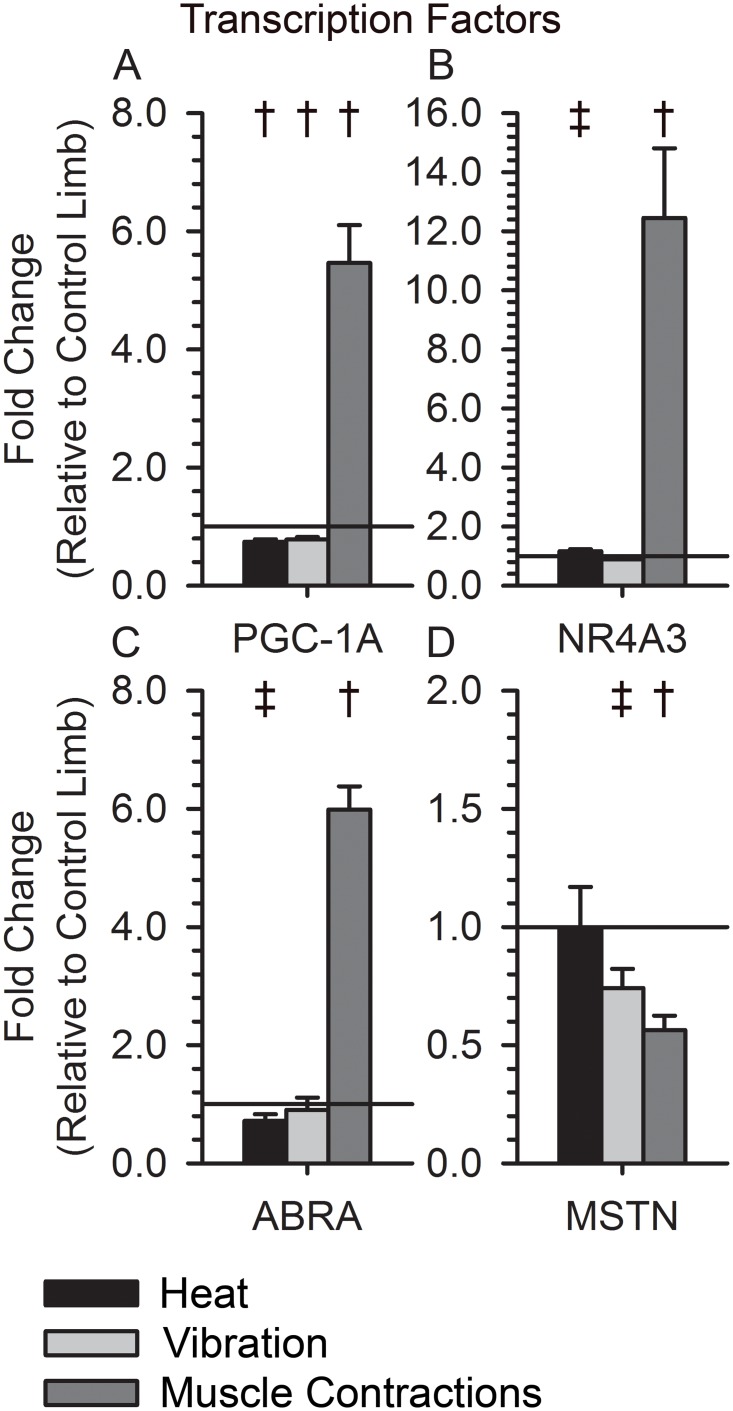
Expression of Transcription Factors following Muscle Contractions, Vibration, and Heat. (A)PGC-1α expression increased after muscle contractions (5.46±0.64, p<0.001) and decreased after vibration and heat(0.78±0.04, p<0.008; 0.74±0.04, p<0.003). (B) NR4A3 expression increased after muscle contractions (12.45±2.36, p<0.001), unchanged after vibration (0.88±0.07, p<0.15), and slightly increased after heat (1.16±0.08, p = 0.09). (C) ABRA expression increased after muscle contractions (5.98±0.40, p<0.001), unchanged after vibration (0.91±0.21, p<0.44), and slightly decreased after heat (0.72±0.11, p<0.07). (D) MSTN expression decreased after muscle contractions (0.56±0.06, p = 0.002), slightly decreased after vibration (0.74±0.08, p = 0.06), and unchanged after heat (1.0±0.17, p = 0.66). † indicates a p-value < 0.05 for a within group paired t-test. ‡ indicates a p-value < 0.10 for a within group paired t-test.

We further examined a subset of 12 genes: mitochondrial pyruvate carrier 2(BRP44), mitochondrial pyruvate carrier 1(BRP44L), mitofusin 1(MFN1), mitofusin 2(MFN2), pyruvate dehydrogenase kinase, isozyme 4(PDK4), pyruvate dehydrogenase alpha 1(PDHA1), pyruvate dehydrogenase beta(PDHB), pyruvate dehydrogenase complex component X(PDHX), PGC-1α, NR4A3, ABRA, and MSTN. These genes have important regulatory and enzymatic roles in skeletal muscle mitochondrial function (PCG-1α, BRP44, BRP44L, MFN1, MFN2), glucose metabolism (NR4A3, PDK4, PDHA1, PDHB, PDHX), and muscle hypertrophy (ABRA, MSTN). Muscle contractions up regulated the expression of the potent transcription factors PGC-1α, NR4A3, and ABRA, and down regulated MSTN (Fig 4; p < 0.05). Vibration down regulated PGC-1α and MSTN, with a limited change in the expression of NR4A3 and ABRA (Fig 4; p < 0.05). Heat down regulated the expression of PGC-1α, NR4A3, and ABRA, but did not change MSTN expression (Fig 4: p < 0.05).

**Fig 5:**
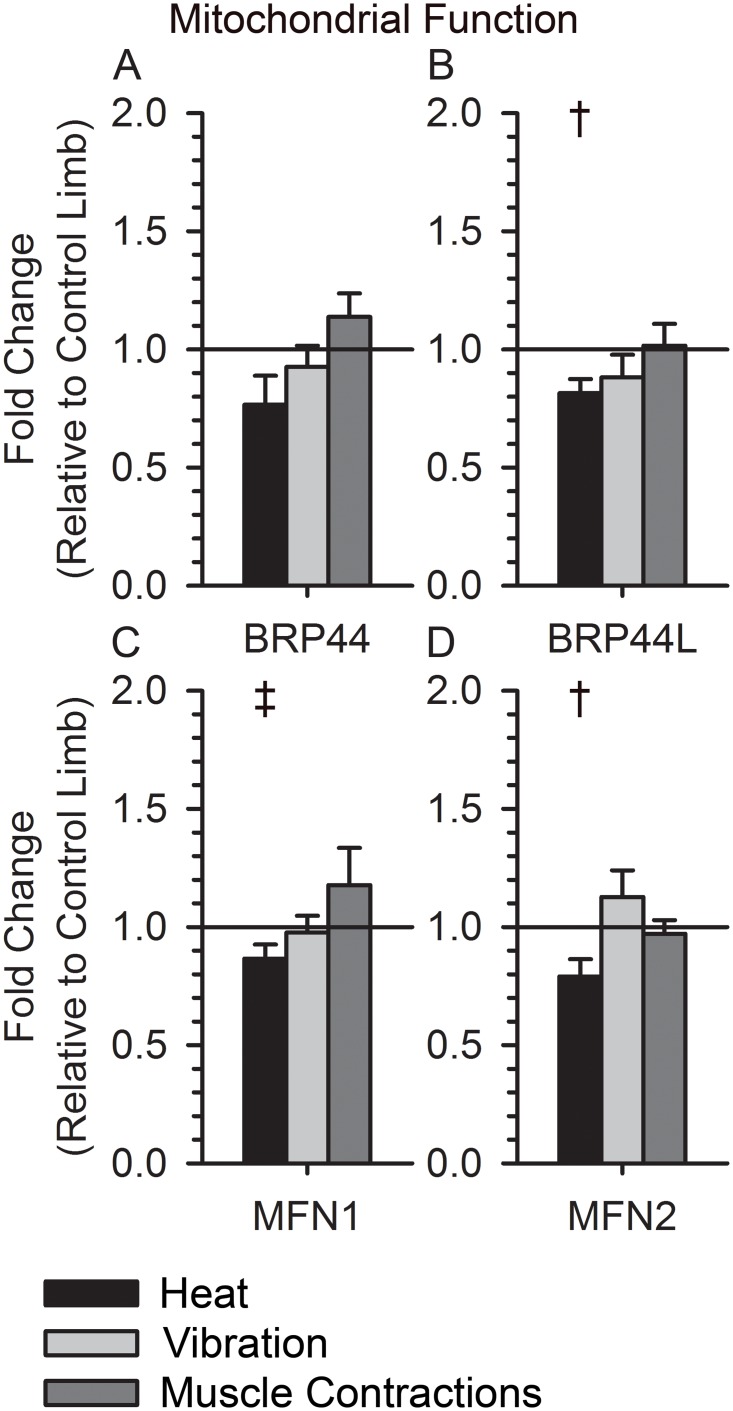
Expression of Mitochondrial Genes following Muscle Contractions, Vibration, and Heat. (A) BRP44 expression was unchanged after muscle contractions and vibration (1.14±0.10, p = 0.25; 0.93±0.09, p = 0.42) and decreased in most participants after heat (0.77±0.12, p = 0.12). (B) BRP44L expression was unchanged after muscle contractions and vibration (1.02±0.09, p = 0.97; 0.88±0.10, p = 0.27) and decreased after heat (0.82±0.06, p = 0.03). (C) MFN1 expression was unchanged after muscle contractions and vibration (1.18±0.16, p = 0.42; 0.98±0.07, p = 0.67), and slightly decreased after heat (0.87±0.06, p = 0.07). (D) MFN2 expression was unchanged after muscle contractions and vibration (0.97±0.06, p = 0.54; 1.13±0.11, p = 0.44) and slightly decreased after heat (0.79±0.07, p = 0.05). † indicates a p-value < 0.05 for a within group paired t-test. ‡ indicates a p-value < 0.10 for a within group paired t-test.

**Fig 6:**
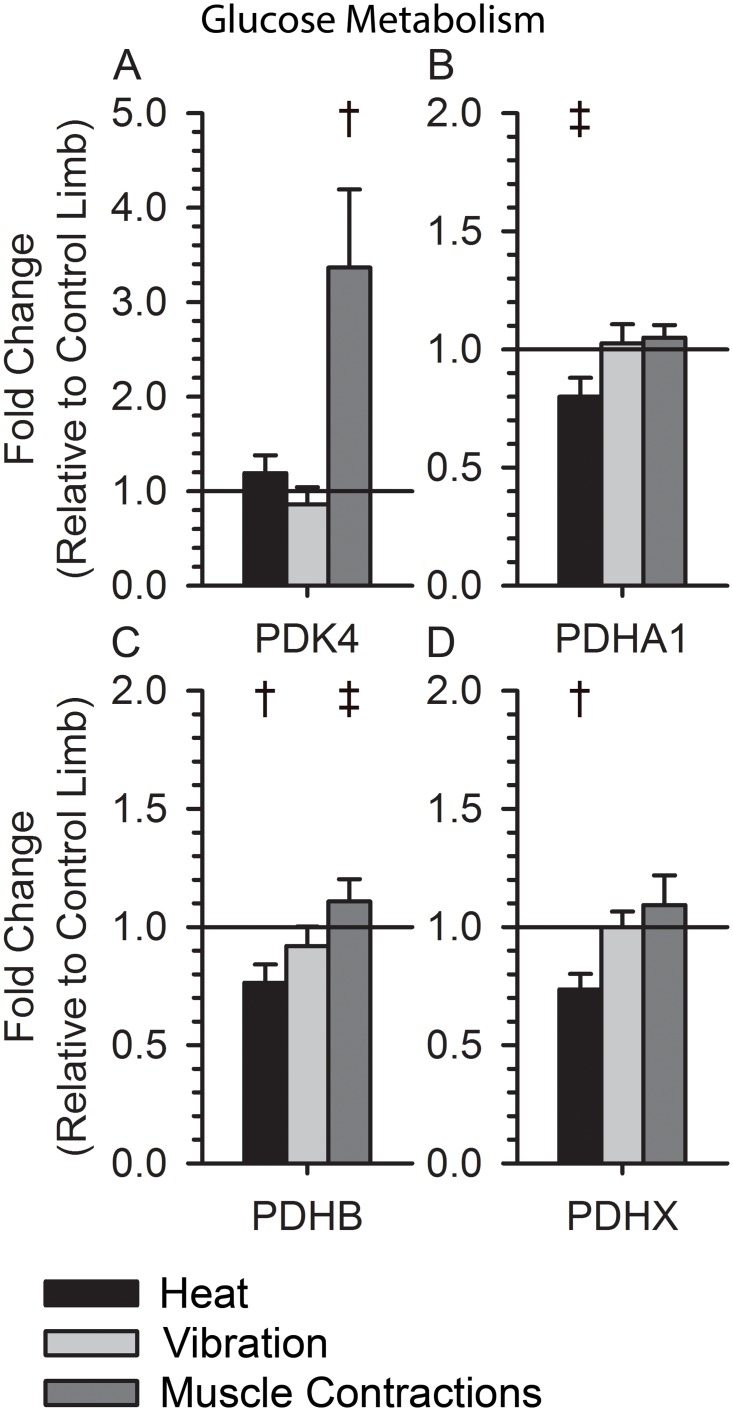
Expression of Glucose Metabolism Genes following Muscle Contractions, Vibration, and Heat. (A) PDK4 expression was increased after muscle contractions (3.37±0.83, p<0.008) and unchagned after vibration and heat (0.86±0.18, p = 0.36; 1.19±0.19, p = 0.67). (B) PDHA1 expression was unchanged after muscle contractions and vibration (1.05±0.05, p = 0.46; 1.03±0.08, p = 0.90) and decreased after heat (0.80±0.08, p = 0.06). (C) PDHB expression was slightly increased after muscle contractions(1.11±0.09, p = 0.35), unchanged after vibration (0.92±0.08, p = 0.36), and decreased after heat (0.76±0.08, p = 0.04). (D) PDHX expression was unchanged after muscle contractions and vibration (1.09±0.12, p = 0.59; 1.0±0.07, p = 0.88) and decreased after heat (0.74±0.07, p = 0.02). † indicates a p-value < 0.05 for a within group paired t-test. ‡ indicates a p-value < 0.10 for a within group paired t-test.

Heat Stress induced a significant down regulation of genes important for mitochondrial function (BRP44, BRP44L, MFN1, MFN2; p < 0.05), but neither vibration nor muscle contractions altered their expression (Fig 5). Additionally, heat down regulated genes used during glucose metabolism (PDHA1, PDHB, and PDHX; p < 0.05), whereas muscle contractions increased the expression of PDK4 (Fig 6; p < 0.05). Vibration did not consistently alter the signaling of genes related to glucose metabolism (Fig 6).

We used RT-qPCR to verify that ABRA, ANKRD, and NR4A3 responded similar under the heat and active contraction stresses. Specifically, ABRA was 0.96 ± 0.26 and 11.75 ± 2.03 after heat and active contractions, respectively, which was even more robust than the array findings. The ANKRD1 gene was 0.70 ± 0.38 and 12.56 ± 4.27 after heat and active contractions, respectively, which again corroborated our array findings. Lastly, the NR4A3 was 3.35 ± 4.99 and 217.71 ± 132.03 after heat and active contractions, respectively, verifying some of the main findings of the gene array.

## Discussion

A defined dose of active stress (muscle contractions), passive mechanical stress (vibration), and systemic heat stress induced: 1) significant muscle fatigue, 2) a purely mechanical stress independent of muscle activation, and 3) activated a significant systemic sympathetic response, respectively. Accordingly, this study created a novel opportunity to compare the effects of each stressor on skeletal muscle gene regulation.

Skeletal muscle contractions increased genes associated with the regulation of oxidative metabolism, mitochondrial function, and muscle hypertrophy and repressed muscle atrophy genes, consistent with previous reports [8, 9, 17, 36–40]. However, our new findings support that vibration regulated a unique "load sensing” pathway (Wnt) and a well-known muscle hypertrophy pathway (MSTN); while whole-body heat stress exclusively regulated a major “muscle remodeling” transcription factor (ANKRD1). These findings demonstrate, for the first time, that each stressor regulates a unique gene expression signature in skeletal muscle. These findings raise the possibility that a “blend” of stressors may be a novel strategy and futuristic approach to optimize skeletal muscle tissue health; in particular in people with disability who cannot always voluntarily drive their own skeletal muscle.

### Skeletal Muscle Response to Vibration

By designing a method to transmit a purely mechanical load (vibration), we were able to ascertain the influence of mechanical signaling, without muscle activity, on skeletal muscle pathways. Overall, the dose of vibration used in this study regulated fewer genes as compared to active skeletal muscle contraction. However, there was overlap in genes regulated by both vibration and muscle activation. Specifically, a single dose of vibration suppressed MSTN, a well-established muscle atrophy regulator (fostering hypertrophy). Using a 30 Hz frequency for vibration, investigators showed a similar down regulation of MSTN in the mouse using both an in vivo and in vitro (muscle cell culture) analysis [19]. However, Ceccarelli and colleagues did not compare the magnitude of the regulation induced by vibration to active muscle exercise. In our study, active muscle exercise repressed MSTN to a greater degree than that of vibration. Based on the findings from this study, regulating the MSTN gene would be most effective if the stress was induced through active muscle exercise rather than using a vibration plate. However, if the individual is unable to activate their skeletal muscle (CNS injury) or they are restricted from activating the muscle (fracture, surgery, ligament repair), vibration may provide an alternative method to regulate MSTN and potentially attenuate skeletal muscle atrophy.

Vibration up regulated a major transcription factor gene (FOXK2). Interestingly, active muscle exercise and heat stress did not up regulate FOXK2. FOXK2 modulates the Wnt pathway, which is known to be associated with myogenesis, muscle fiber type differentiation, and satellite cell recruitment after injury [41–43]. The FOXK2 gene supports the nuclear translocation of the disheveled (DVL) protein that then regulates the transcription of Wnt signaling receptors [43]. Increased FOXK2 expression after passive vibration may indicate a potential downstream increase of DVL and Wnt signaling receptor activity, particularly with repeat exposures to the vibration stress. To our knowledge, no previous reports have examined the Wnt pathway in response to localized skeletal muscle vibration in humans. The finding that the FOXK2 gene is regulated exclusively by vibration underscores the need for futures studies designed to examine the downstream effects of FOXK2 on skeletal muscle function.

Indeed, passive vibration may offer a unique mechanical stimulus to influence the skeletal muscle phenotype. In order to compare vibration to active muscle contraction, it is important to de-couple the mechanical event from the active contraction of the skeletal muscle, as we have done in this study [35]. Previous studies with whole body vibration induce active muscle contraction via the vestibular system in response to the oscillatory mechanical frequencies sensed at the level of the head [44–46]. By de-coupling these mechanical signals, we were able to contrast, for the first time, the effects of pure vibration with active muscle contraction on gene regulation in humans.

### Skeletal Muscle Response to Heat Stress

The dose of whole body heat stress used in this study increased heart rate and core body temperature [27], indicative of a passive systemic stress to the sympathetic nervous system. An intriguing finding was that whole-body heat stress suppressed the expression of PGC-1α, the exact opposite of the over 5-fold increase observed with active muscle contraction. PGC-1α is a well-known regulator of mitochondrial biogenesis in healthy skeletal muscle [47]. PGC-1α is down regulated in skeletal muscle in people with long-standing diabetes [48] and correlated with a shift from an oxidative muscle to a glycolytic muscle [49, 50]. The short-term suppression of PGC-1α because of heat stress may indicate a shift in substrate utilization at the skeletal muscle level. In support of this view, skeletal muscle blood flow decreases and shunts the blood to the skin capillary beds to attenuate core body temperature [51, 52]. The skeletal muscle is not performing work during heat stress and the increased sympathetic drive and catecholamines support enhanced gluconeogenesis in the liver. The increased blood glucose availability combined with the decrease skeletal muscle work demand may explain the acute down regulation of PGC-1α.

Recent work in our lab and others [53] support that blood glucose increases after passive whole body heat stress. Thus, whole body heat stress may trigger the exact opposite of what typically occurs with long duration exercise. That is, during exercise, an increase in PGC-1α regulation inhibits glucose oxidation to favor fatty acid oxidation and glycogen synthesis [37]. Conversely, during heat stress, there is a decreased need for ATP in the skeletal muscle that may trigger a less efficient energy utilization strategy. This may explain the acute suppression of PGC-1α, the suppression of the mitochondrial protein carrier (BRP44L), and the suppression of pyruvate dehydrogenase genes (PDHA1, PDHB, and PDHX) that we observed after heat. We speculate that a shift in substrate utilization may trigger a more suitable environment for muscle repair after muscle damage from exercise or disease processes. Skeletal muscle repair, including proliferation of satellite cells, may depend on the online energy utilization state of the skeletal muscle [54–56]. The duration and intensity of heat stress (dose) on skeletal muscle recovery after injury are important areas for continued examination.

Whole body heat stress significantly repressed the ANKRD1 gene in direct opposition to the findings that active muscle exercise induces the ANKRD1 gene [57, 58]. ANKRD1 is a transcriptional mediator of cellular pathways involved in muscle cell homeostasis [57]. ANKRD1 is elevated following exercise, during muscle regeneration, tissue injury, and several congenital myopathies and muscular dystrophies [57]. ANKRD1 acts as a negative feedback regulator of TNFα induced inflammation by inhibiting NFκB transcription [59], which can also stimulate skeletal muscle disuse atrophy [60]. To our knowledge, no study has previously identified a whole body stressor that acutely suppresses ANKRD1 in skeletal muscle. The potential downstream effects of ANKRD1 suppression are unknown in skeletal muscle. However, ANKRD1 gene suppression may increase a critical acute inflammatory response that could affect muscle regeneration by increased satellite cell recruitment. The relationship between energy substrate use (glycolysis), PGC-1α, and muscle regeneration via satellite cells [54–56], is an important area of continued investigation.

### Methodological and Clinical Considerations

In this study, we isolated a dose of stress to a human leg segment and analyzed the gene regulation. It is important to note that we did not examine whole body aerobic exercise in this study. It may be that whole body exercise involves a natural combination of skeletal muscle signaling associated with heat stress (increased sympathetic drive), active muscle contraction, and vibration. Accordingly, the “net gain” in gene regulation during whole body exercise may represent a combination of all three of the stressors examined in this study. The novelty of this study is that we were able to de-couple each form of stress in an effort to understand the specific signaling to skeletal muscle.

While this study discovered several new findings, it is not without several important methodological considerations. First, we based our dose of stress on previous basic science investigations while also weighing the importance of feasibility for human subjects. Second, we have a limited number of participants with each stressor in this study, however, our within subject control provided offered excellent power. Finally, we studied muscle with fast contractile speeds from both paralyzed and non-paralyzed subjects, but from two different muscles (soleus and VL). For safety reasons, people with SCI could not tolerate heat, thus, we evaluated the VL in healthy humans. We chose the VL in healthy subjects because, based on our pilot studies, the VL most closely resembled the gene expression of the paralyzed soleus muscle (please see pilot data in methods). Taken together, the findings from this study are informative and robust, but must be interpreted carefully given the methodological considerations.

### Summary and Conclusions

This study showed that active muscle contractions induced gene expression associated with metabolism, including a large up regulation of PGC-1α and down regulation of MSTN. Vibration similarly caused a down regulation of MSTN, but to a lesser extent than the change observed with active muscle contraction. Vibration exclusively up regulated FOXK2, a gene associated with the Wnt pathway. Heat stress down regulated PGC-1α and ANKRD1 genes. These findings suggest a different substrate use from that of exercise and a potential up regulation of muscle regeneration through satellite cell activation. Overall, this study provides novel findings regarding the responsiveness of human skeletal muscle signaling to mechanical, physiological, and environmental stress. Understanding optimal methods to support skeletal muscle health, using non-pharmacologic interventions, will be instrumental in identifying new regenerative medicine rehabilitation protocols in the future.

## References

[1] Luzi L. Human Evolution and Physical Exercise: The Concept of Being “Born to Run” In: Luzi L, editor. Cellular Physiology and Metabolism of Physical Exercise: Springer Milan; 2012 p. 1–7.

[2] Zhorne R, Dudley-Javoroski S, Shields RK. Skeletal muscle activity and CNS neuro-plasticity. Neural Regen Res. 2016;11(1):69–70. 10.4103/1673-5374.169623 26981083 PMC4774230

[3] Duckworth WC, Solomon SS, Jallepalli P, Heckemeyer C, Finnern J, Powers A. Glucose intolerance due to insulin resistance in patients with spinal cord injuries. Diabetes. 1980;29(11):906–10. Epub 1980/11/01. . 7429029 10.2337/diab.29.11.906

[4] Dudley-Javoroski S, Shields RK. Muscle and bone plasticity after spinal cord injury: review of adaptations to disuse and to electrical muscle stimulation. Journal of rehabilitation research and development. 2008;45(2):283–96. Epub 2008/06/21. 18566946 10.1682/jrrd.2007.02.0031 PMC2744487

[5] Lavela SL, Weaver FM, Goldstein B, Chen K, Miskevics S, Rajan S, et al Diabetes mellitus in individuals with spinal cord injury or disorder. J Spinal Cord Med. 2006;29(4):387–95. Epub 2006/10/19. 17044389 10.1080/10790268.2006.11753887 PMC1864854

[6] Wilmet E, Ismail AA, Heilporn A, Welraeds D, Bergmann P. Longitudinal study of the bone mineral content and of soft tissue composition after spinal cord section. Paraplegia. 1995;33(11):674–7. Epub 1995/11/01. 10.1038/sc.1995.141 . 8584304

[7] Bjornholm M, Zierath JR. Insulin signal transduction in human skeletal muscle: identifying the defects in Type II diabetes. Biochemical Society transactions. 2005;33(Pt 2):354–7. Epub 2005/03/25. 10.1042/BST0330354 . 15787605

[8] Adams CM, Suneja M, Dudley-Javoroski S, Shields RK. Altered mRNA expression after long-term soleus electrical stimulation training in humans with paralysis. Muscle Nerve. 2011;43(1):65–75. Epub 2010/12/21. 10.1002/mus.21831 21171097 PMC3058836

[9] Petrie MA, Suneja M, Faidley E, Shields RK. A minimal dose of electrically induced muscle activity regulates distinct gene signaling pathways in humans with spinal cord injury. PloS one. 2014;9(12):e115791 10.1371/journal.pone.0115791 25531450 PMC4274164

[10] Bauman WA, Spungen AM. Disorders of carbohydrate and lipid metabolism in veterans with paraplegia or quadriplegia: a model of premature aging. Metabolism: clinical and experimental. 1994;43(6):749–56. Epub 1994/06/01. . 8201966 10.1016/0026-0495(94)90126-0

[11] Bauman WA, Spungen AM, Adkins RH, Kemp BJ. Metabolic and endocrine changes in persons aging with spinal cord injury. Assistive technology: the official journal of RESNA. 1999;11(2):88–96. Epub 2000/09/30. 10.1080/10400435.1999.10131993 . 11010069

[12] Buchholz AC, McGillivray CF, Pencharz PB. Physical activity levels are low in free-living adults with chronic paraplegia. Obes Res. 2003;11(4):563–70. 10.1038/Oby.2003.79. ISI:000182195600012. 12690086

[13] Burstein R, Zeilig G, Royburt M, Epstein Y, Ohry A. Insulin resistance in paraplegics—Effect of one bout of acute exercise. International journal of sports medicine. 1996;17(4):272–6. 10.1055/s-2007-972846. ISI:A1996UR26800007. 8814509

[14] Gorgey AS, Mather KJ, Cupp HR, Gater DR. Effects of resistance training on adiposity and metabolism after spinal cord injury. Medicine and science in sports and exercise. 2012;44(1):165–74. Epub 2011/06/11. 10.1249/MSS.0b013e31822672aa . 21659900

[15] Gorgey AS, Mather KJ, Gater DR. Central adiposity associations to carbohydrate and lipid metabolism in individuals with complete motor spinal cord injury. Metabolism: clinical and experimental. 2011;60(6):843–51. Epub 2010/09/28. 10.1016/j.metabol.2010.08.002 . 20870252

[16] McCully KK, Mulcahy TK, Ryan TE, Zhao Q. Skeletal muscle metabolism in individuals with spinal cord injury. J Appl Physiol. 2011;111(1):143–8. Epub 2011/04/23. 10.1152/japplphysiol.00094.2011 21512153 PMC3137532

[17] Petrie M, Suneja M, Shields RK. Low Frequency Stimulation Regulates Metabolic Gene Expression in Paralyzed Muscle. Journal of applied physiology. 2015:jap 00628 2014. 10.1152/japplphysiol.00628.2014 . 25635001 PMC4360022

[18] Petrie MA, Suneja M, Faidley E, Shields RK. Low force contractions induce fatigue consistent with muscle mRNA expression in people with spinal cord injury. Physiol Rep. 2014;2(2):e00248 Epub 2014/04/20. 10.1002/phy2.248 . 24744911 PMC3966256

[19] Ceccarelli G, Benedetti L, Galli D, Pre D, Silvani G, Crosetto N, et al Low-amplitude high frequency vibration down-regulates myostatin and atrogin-1 expression, two components of the atrophy pathway in muscle cells. Journal of tissue engineering and regenerative medicine. 2014;8(5):396–406. 10.1002/term.1533 . 22711460

[20] Xie L, Rubin C, Judex S. Enhancement of the adolescent murine musculoskeletal system using low-level mechanical vibrations. Journal of applied physiology. 2008;104(4):1056–62. 10.1152/japplphysiol.00764.2007 . 18258802

[21] Chang SH, Dudley-Javoroski S, Shields RK. Gravitational force modulates muscle activity during mechanical oscillation of the tibia in humans. J Electromyogr Kinesiol. 2011;21(5):847–53. Epub 2011/06/29. 10.1016/j.jelekin.2011.06.001 21708472 PMC3355375

[22] Chang SH, Tseng SC, McHenry CL, Littmann AE, Suneja M, Shields RK. Limb segment vibration modulates spinal reflex excitability and muscle mRNA expression after spinal cord injury. Clinical neurophysiology: official journal of the International Federation of Clinical Neurophysiology. 2012;123(3):558–68. Epub 2011/10/04. 10.1016/j.clinph.2011.08.001 21963319 PMC3270316

[23] Hamrick MW. A role for myokines in muscle-bone interactions. Exercise and sport sciences reviews. 2011;39(1):43–7. 10.1097/JES.0b013e318201f601 21088601 PMC3791922

[24] Laukkanen T, Khan H, Zaccardi F, Laukkanen JA. Association between sauna bathing and fatal cardiovascular and all-cause mortality events. JAMA Intern Med. 2015;175(4):542–8. 10.1001/jamainternmed.2014.8187 . 25705824

[25] Carrier DR, Kapoor AK, Kimura T, Nickels MK, Satwanti, Scott EC, et al The Energetic Paradox of Human Running and Hominid Evolution [and Comments and Reply]. Current Anthropology. 1984;25(4):483–95.

[26] Seebacher F. Responses to temperature variation: integration of thermoregulation and metabolism in vertebrates. J Exp Biol. 2009;212(18):2885–91. 10.1242/jeb.024430 . 19717669

[27] Iguchi M, Littmann AE, Chang SH, Wester LA, Knipper JS, Shields RK. Heat stress and cardiovascular, hormonal, and heat shock proteins in humans. Journal of athletic training. 2012;47(2):184–90. Epub 2012/04/11. 22488284 10.4085/1062-6050-47.2.184 PMC3418130

[28] Febbraio MA, Ott P, Nielsen HB, Steensberg A, Keller C, Krustrup P, et al Exercise induces hepatosplanchnic release of heat shock protein 72 in humans. J Physiol. 2002;544(Pt 3):957–62. 12411538 10.1113/jphysiol.2002.025148 PMC2290618

[29] Yamada P, Amorim F, Moseley P, Schneider S. Heat shock protein 72 response to exercise in humans. Sports Med. 2008;38(9):715–33. . 18712940 10.2165/00007256-200838090-00002

[30] Fehrenbach E, Niess AM, Voelker K, Northoff H, Mooren FC. Exercise intensity and duration affect blood soluble HSP72. International journal of sports medicine. 2005;26(7):552–7. 10.1055/s-2004-830334 . 16195988

[31] Walsh RC, Koukoulas I, Garnham A, Moseley PL, Hargreaves M, Febbraio MA. Exercise increases serum Hsp72 in humans. Cell Stress Chaperones. 2001;6(4):386–93. 11795476 10.1379/1466-1268(2001)006<0386:eishih>2.0.co;2 PMC434422

[32] Luden N, Minchev K, Hayes E, Louis E, Trappe T, Trappe S. Human vastus lateralis and soleus muscles display divergent cellular contractile properties. American journal of physiology Regulatory, integrative and comparative physiology. 2008;295(5):R1593–8. 10.1152/ajpregu.90564.2008 18815206 PMC2584861

[33] Ericson MO, Nisell R, Ekholm J. Quantified electromyography of lower-limb muscles during level walking. Scandinavian journal of rehabilitation medicine. 1986;18(4):159–63. . 3810082

[34] Shields RK. Fatigability, relaxation properties, and electromyographic responses of the human paralyzed soleus muscle. Journal of neurophysiology. 1995;73(6):2195–206. Epub 1995/06/01. . 7666132 10.1152/jn.1995.73.6.2195

[35] McHenry CL, Wu J, Shields RK. Potential regenerative rehabilitation technology: implications of mechanical stimuli to tissue health. BMC Res Notes. 2014;7:334 10.1186/1756-0500-7-334 24894666 PMC4055276

[36] Egan B, Carson BP, Garcia-Roves PM, Chibalin AV, Sarsfield FM, Barron N, et al Exercise intensity-dependent regulation of peroxisome proliferator-activated receptor coactivator-1 mRNA abundance is associated with differential activation of upstream signalling kinases in human skeletal muscle. J Physiol. 2010;588(Pt 10):1779–90. Epub 2010/03/24. 10.1113/jphysiol.2010.188011 20308248 PMC2887994

[37] Finck BN, Kelly DP. PGC-1 coactivators: inducible regulators of energy metabolism in health and disease. The Journal of clinical investigation. 2006;116(3):615–22. Epub 2006/03/03. 10.1172/JCI27794 16511594 PMC1386111

[38] Irrcher I, Hood DA. Regulation of Egr-1, SRF, and Sp1 mRNA expression in contracting skeletal muscle cells. Journal of applied physiology. 2004;97(6):2207–13. Epub 2004/08/18. 10.1152/japplphysiol.00388.2004 . 15310743

[39] Little JP, Safdar A, Bishop D, Tarnopolsky MA, Gibala MJ. An acute bout of high-intensity interval training increases the nuclear abundance of PGC-1alpha and activates mitochondrial biogenesis in human skeletal muscle. American journal of physiology Regulatory, integrative and comparative physiology. 2011;300(6):R1303–10. Epub 2011/04/01. 10.1152/ajpregu.00538.2010 . 21451146

[40] Wallace MA, Hock MB, Hazen BC, Kralli A, Snow RJ, Russell AP. Striated muscle activator of Rho signalling (STARS) is a PGC-1alpha/oestrogen-related receptor-alpha target gene and is upregulated in human skeletal muscle after endurance exercise. J Physiol. 2011;589(Pt 8):2027–39. Epub 2011/04/14. 10.1113/jphysiol.2011.205468 21486805 PMC3090601

[41] Cisternas P, Henriquez JP, Brandan E, Inestrosa NC. Wnt signaling in skeletal muscle dynamics: myogenesis, neuromuscular synapse and fibrosis. Mol Neurobiol. 2014;49(1):574–89. 10.1007/s12035-013-8540-5 . 24014138

[42] Rudnicki MA, Williams BO. Wnt signaling in bone and muscle. Bone. 2015;80:60–6. 10.1016/j.bone.2015.02.009 26453496 PMC4600531

[43] Wang W, Li X, Lee M, Jun S, Aziz KE, Feng L, et al FOXKs promote Wnt/beta-catenin signaling by translocating DVL into the nucleus. Developmental cell. 2015;32(6):707–18. 10.1016/j.devcel.2015.01.031 25805136 PMC4374128

[44] Rittweger J. Vibration as an exercise modality: how it may work, and what its potential might be. European journal of applied physiology. 2010;108(5):877–904. 10.1007/s00421-009-1303-3 . 20012646

[45] Item F, Nocito A, Thony S, Bachler T, Boutellier U, Wenger RH, et al Combined whole-body vibration, resistance exercise, and sustained vascular occlusion increases PGC-1alpha and VEGF mRNA abundances. European journal of applied physiology. 2013;113(4):1081–90. 10.1007/s00421-012-2524-4 . 23086295

[46] Pietrangelo T, Mancinelli R, Toniolo L, Cancellara L, Paoli A, Puglielli C, et al Effects of local vibrations on skeletal muscle trophism in elderly people: mechanical, cellular, and molecular events. Int J Mol Med. 2009;24(4):503–12. . 19724891 10.3892/ijmm_00000259

[47] Chan MC, Arany Z. The many roles of PGC-1alpha in muscle—recent developments. Metabolism: clinical and experimental. 2014;63(4):441–51. Epub 2014/02/25. 10.1016/j.metabol.2014.01.006 . 24559845 PMC4040247

[48] Mootha VK, Lindgren CM, Eriksson KF, Subramanian A, Sihag S, Lehar J, et al PGC-1alpha-responsive genes involved in oxidative phosphorylation are coordinately downregulated in human diabetes. Nature genetics. 2003;34(3):267–73. Epub 2003/06/17. 10.1038/ng1180 . 12808457

[49] Stuart CA, McCurry MP, Marino A, South MA, Howell ME, Layne AS, et al Slow-twitch fiber proportion in skeletal muscle correlates with insulin responsiveness. The Journal of clinical endocrinology and metabolism. 2013;98(5):2027–36. 10.1210/jc.2012-3876 23515448 PMC3644602

[50] Sun Z, Liu L, Liu N, Liu Y. Muscular response and adaptation to diabetes mellitus. Front Biosci. 2008;13:4765–94. . 18508544 10.2741/3038

[51] Edholm OG, Fox RH, Macpherson RK. The effect of body heating on the circulation in skin and muscle. J Physiol. 1956;134(3):612–9. 13398947 10.1113/jphysiol.1956.sp005669 PMC1359165

[52] Smolander J, Kolari P. Laser-Doppler and plethysmographic skin blood flow during exercise and during acute heat stress in the sauna. Eur J Appl Physiol Occup Physiol. 1985;54(4):371–7. . 2933255 10.1007/BF02337180

[53] Dumke CL, Slivka DR, Cuddy JS, Hailes WS, Rose SM, Ruby BC. The Effect of Environmental Temperature on Glucose and Insulin After an Oral Glucose Tolerance Test in Healthy Young Men. Wilderness Environ Med. 2015;26(3):335–42. 10.1016/j.wem.2015.03.002 . 25937547

[54] Lunt SY, Vander Heiden MG. Aerobic glycolysis: meeting the metabolic requirements of cell proliferation. Annu Rev Cell Dev Biol. 2011;27:441–64. 10.1146/annurev-cellbio-092910-154237 . 21985671

[55] Vander Heiden MG, Locasale JW, Swanson KD, Sharfi H, Heffron GJ, Amador-Noguez D, et al Evidence for an alternative glycolytic pathway in rapidly proliferating cells. Science. 2010;329(5998):1492–9. 10.1126/science.1188015 20847263 PMC3030121

[56] Vander Heiden MG, Lunt SY, Dayton TL, Fiske BP, Israelsen WJ, Mattaini KR, et al Metabolic pathway alterations that support cell proliferation. Cold Spring Harb Symp Quant Biol. 2011;76:325–34. 10.1101/sqb.2012.76.010900 . 22262476

[57] Kojic S, Radojkovic D, Faulkner G. Muscle ankyrin repeat proteins: their role in striated muscle function in health and disease. Critical reviews in clinical laboratory sciences. 2011;48(5–6):269–94. Epub 2011/12/22. 10.3109/10408363.2011.643857 . 22185618

[58] Mohamed JS, Boriek AM. Loss of desmin triggers mechanosensitivity and up-regulation of Ankrd1 expression through Akt-NF-kappaB signaling pathway in smooth muscle cells. Faseb J. 2012;26(2):757–65. 10.1096/fj.10-160291 . 22085644

[59] Liu XH, Bauman WA, Cardozo C. ANKRD1 modulates inflammatory responses in C2C12 myoblasts through feedback inhibition of NF-kappaB signaling activity. Biochem Biophys Res Commun. 2015;464(1):208–13. 10.1016/j.bbrc.2015.06.118 . 26102030

[60] Jackman RW, Cornwell EW, Wu CL, Kandarian SC. Nuclear factor-kappaB signalling and transcriptional regulation in skeletal muscle atrophy. Exp Physiol. 2013;98(1):19–24. 10.1113/expphysiol.2011.063321 22848079 PMC3505235

